# Heterogeneity of mesenchymal stem cell-derived extracellular vesicles is highly impacted by the tissue/cell source and culture conditions

**DOI:** 10.1186/s13578-022-00786-7

**Published:** 2022-05-02

**Authors:** Ciarra Almeria, Sebastian Kreß, Viktoria Weber, Dominik Egger, Cornelia Kasper

**Affiliations:** 1 Institute of Cell and Tissue Culture Technologies, University of Natural Resources and Life Sciences, Muthgasse 18, 1190 Vienna, Austria; 2 Center for Biomedical Technology, Department for Biomedical Research, Danube University, Dr.-Karl-Dorrek-Straße 30, 3500 Krems an der Donau, Austria

**Keywords:** Extracellular vesicles, Mesenchymal Stem Cells, Cell Culture Conditions, Scalability, Regenerative Medicine

## Abstract

Extracellular vesicles (EVs) are cell-derived membrane structures exerting major effects in physiological as well as pathological processes by functioning as vehicles for the delivery of biomolecules to their target cells. An increasing number of effects previously attributed to cell-based therapies have been recognized to be actually mediated by EVs derived from the respective cells, suggesting the administration of purified EVs instead of living cells for cell-based therapies. In this review, we focus on the heterogeneity of EVs derived from mesenchymal stem/stromal cells (MSC) and summarize upstream process parameters that crucially affect the resulting therapeutic properties and biological functions. Hereby, we discuss the effects of the cell source, medium composition, 3D culture, bioreactor culture and hypoxia. Furthermore, aspects of the isolation and storage strategies influences EVs are described. Conclusively, optimization of upstream process parameters should focus on controlling MSC-derived EV heterogeneity for specific therapeutic applications.

**Graphical Abstract:**

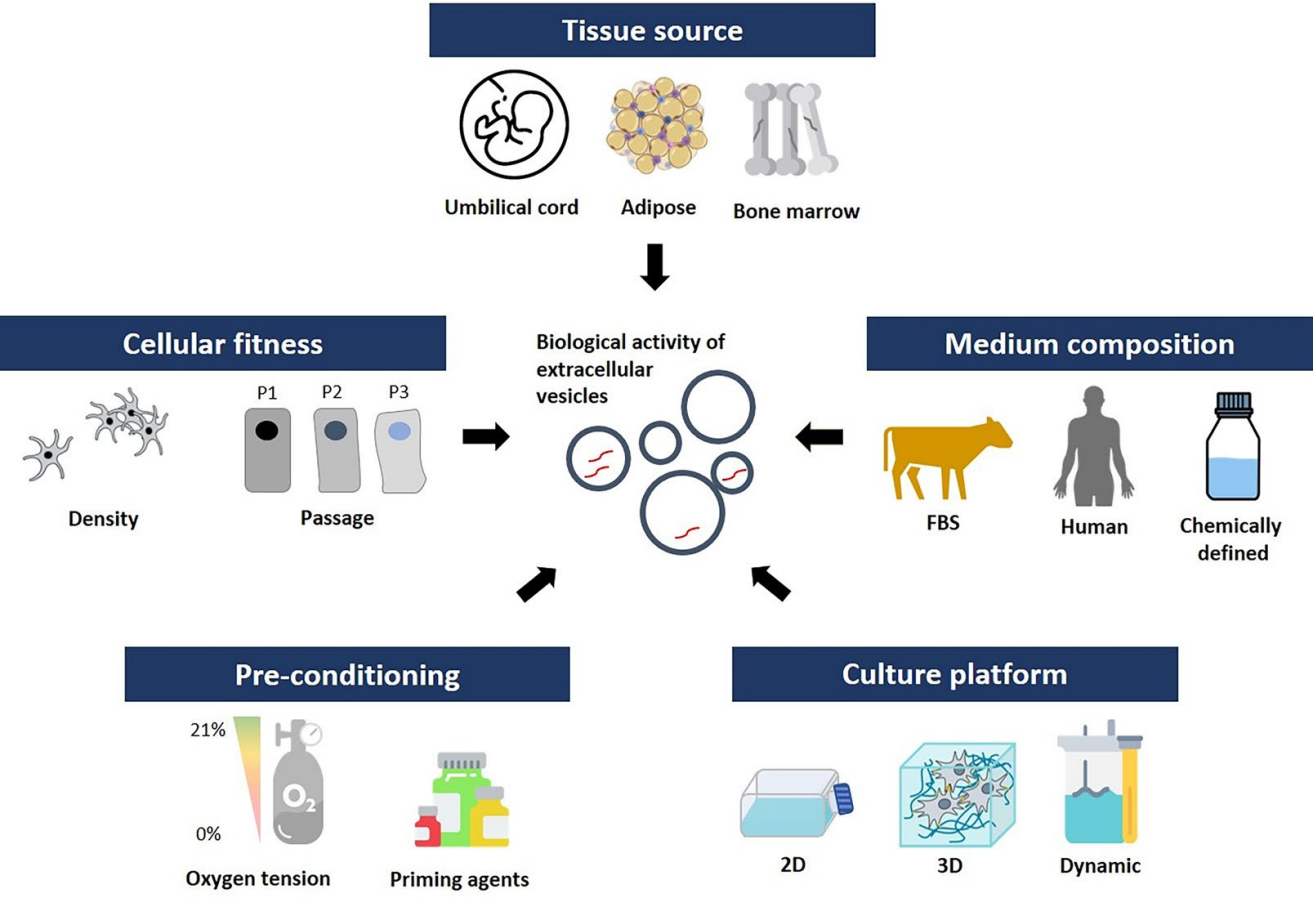

## Introduction

Intercellular communication has long been attributed to soluble factors and adhesion molecules, mediating cell-to-cell interactions. In the last decade, the biological significance of extracellular vesicles (EVs) has gained much recognition, especially their functions in intercellular communication in both, physiological and pathological settings [1].

**Fig. 1:**
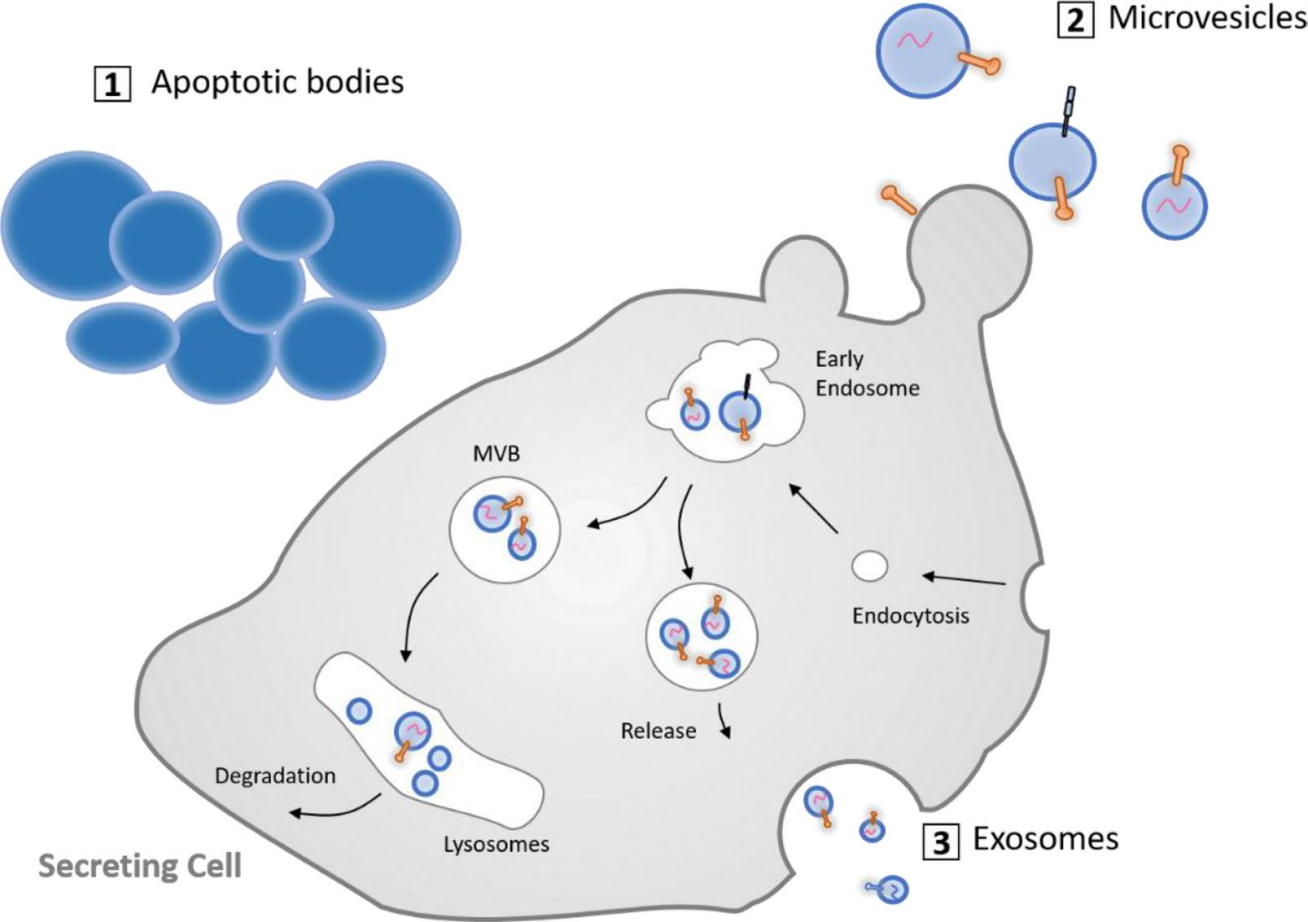
Subtypes of extracellular vesicles in eukaryotic cells. Cells can release three different types of EVs: (i) apoptotic bodies are generated during programmed cell death by membrane blebbing, (ii) microvesicles are shed by outward budding and fission of the plasma membrane, and (iii) exosomes are formed as intraluminal vesicles via inward budding of early endosomes, giving rise to multivesicular bodies (MVBs), which either fuse with lysosomes or with the plasma membrane, leading to the secretion of exosomes. Illustration adapted from Gustafson et al. [7]

Multiple types of EVs have been described with different sites of cellular origin (reviewed in van der Pol et al. [2]) and with distinct molecular and biological properties. Three major EV subtypes (Fig. 1) have been classified based on their size and biogenesis, namely (i) exosomes (40–150 nm in diameter), (ii) microvesicles (100–1000 nm), and (iii) apoptotic bodies (> 1000 nm). Exosomes represent the most extensively studied EV species, and their secretion was originally described as a process that can complement and supplement lysosomal and proteasomal degradation for the removal of obsolete membrane and cytosolic materials [3]. They are formed by the intraluminal invagination of the membrane of the late endosome/multi-vesicular body (MVB) and subsequent fusion of MVBs with the plasma membrane (reviewed in Kreimer et al.  [4] and van der Pol et al. [5]). Microvesicles derive from the plasma membrane and are continuously released from the cell membrane of apparently all cells under physiological conditions, although their release can be further triggered under pathological conditions [6]. Apoptotic bodies, finally, result from the disassembly of apoptotic cells into subcellular fragments. The formation of apoptotic bodies can promote efficient removal of cell debris by means of macrophages, and they were previously regarded as “sealed containers” for substances from dying cells, until the discovery that they are capable of delivering their cargo to healthy recipient cells, as well.

**Fig. 2:**
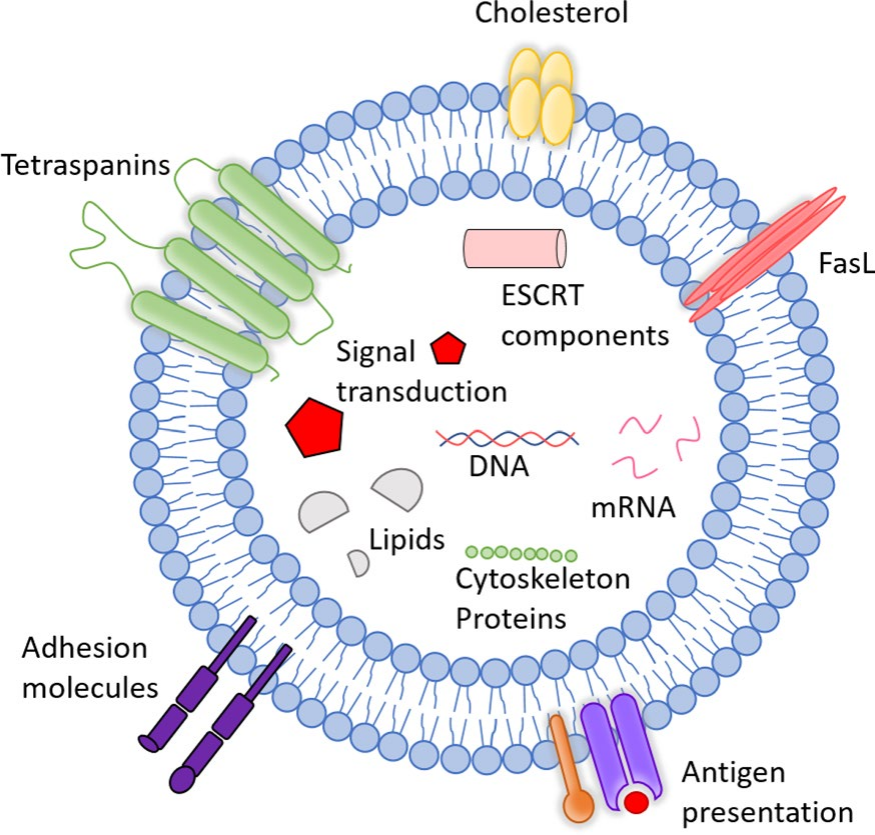
Composition of extracellular vesicles (EVs). EVs are lipid-bound vesicles secreted by most cells into the extracellular space. They consist of lipids, nucleic acids, and proteins, which are specifically associated with the plasma membrane, cytosol, and those related to lipid metabolism of the parent cell. The cargo of EVs can be transferred to target cells and induce biological effects that alter cell behavior

The function of EVs is closely linked to their cargo (Fig. 2), which can include functional mRNA, miRNA, lipids, and proteins. Transfer of this cargo to adjacent or distant recipient cells makes EVs important messengers in cell–cell communication. Beyond their cargo, EV surface molecules are of critical functional significance as they (i) establish connections with the surrounding milieu and with cells, (ii) determine EV mobility, (iii) mediate cellular uptake, (iv) affect immune recognition of EVs by the innate and adaptive immune systems, and (v) may represent effector molecules (such as FasL) [8, 9]. Moreover, these EV surface molecules enable the identification, affinity isolation, and molecular classification of EVs and their use as biomarkers [10].

## Therapeutic potential of mesenchymal stem cell-derived extracellular vesicles

**Fig. 3:**
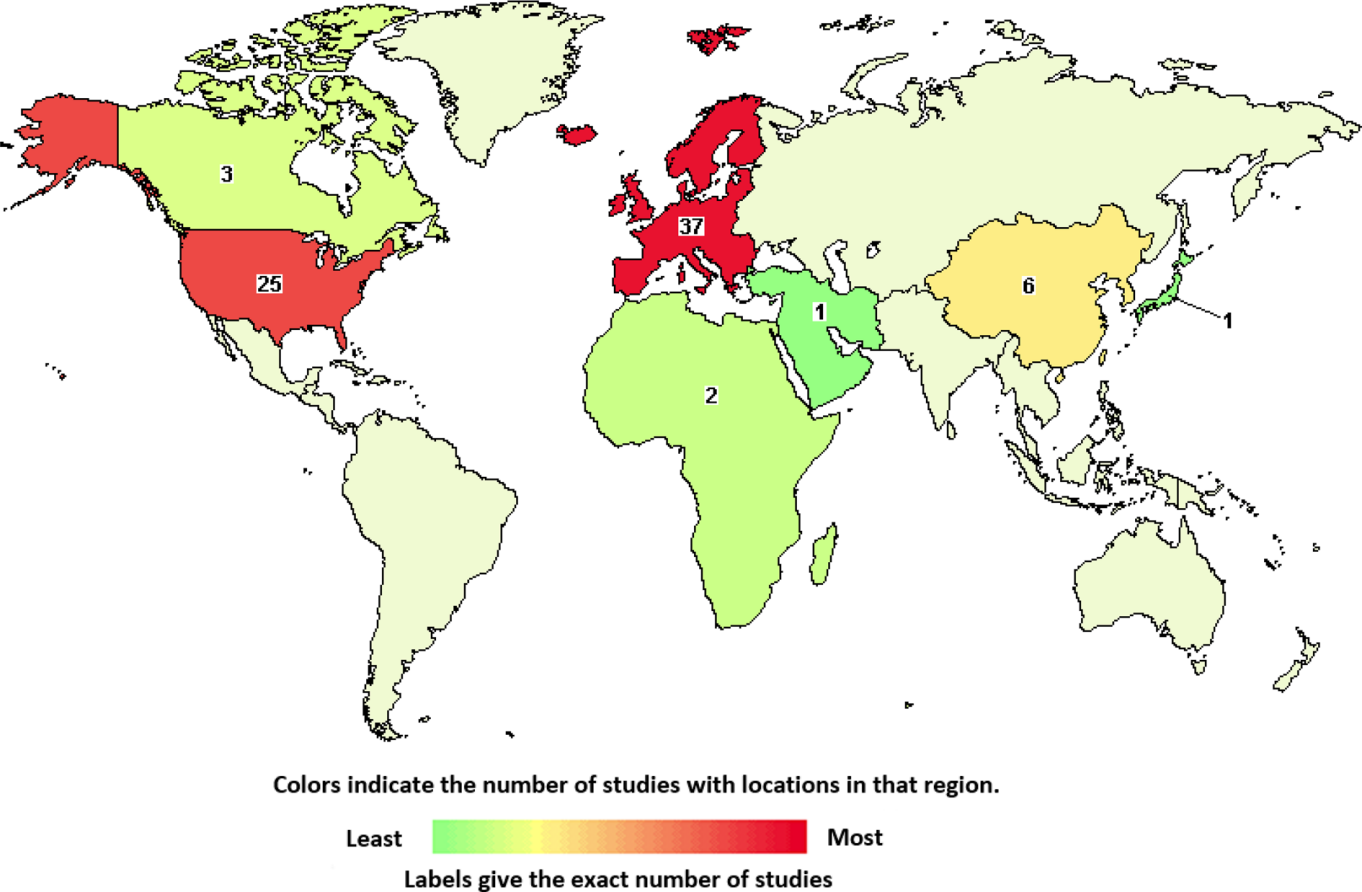
Global clinical trials on extracellular vesicles for cell-free therapy. World map indicating the number of clinical trials registered globally to date (September 2021, search term: extracellular vesicles)

**Fig. 4:**
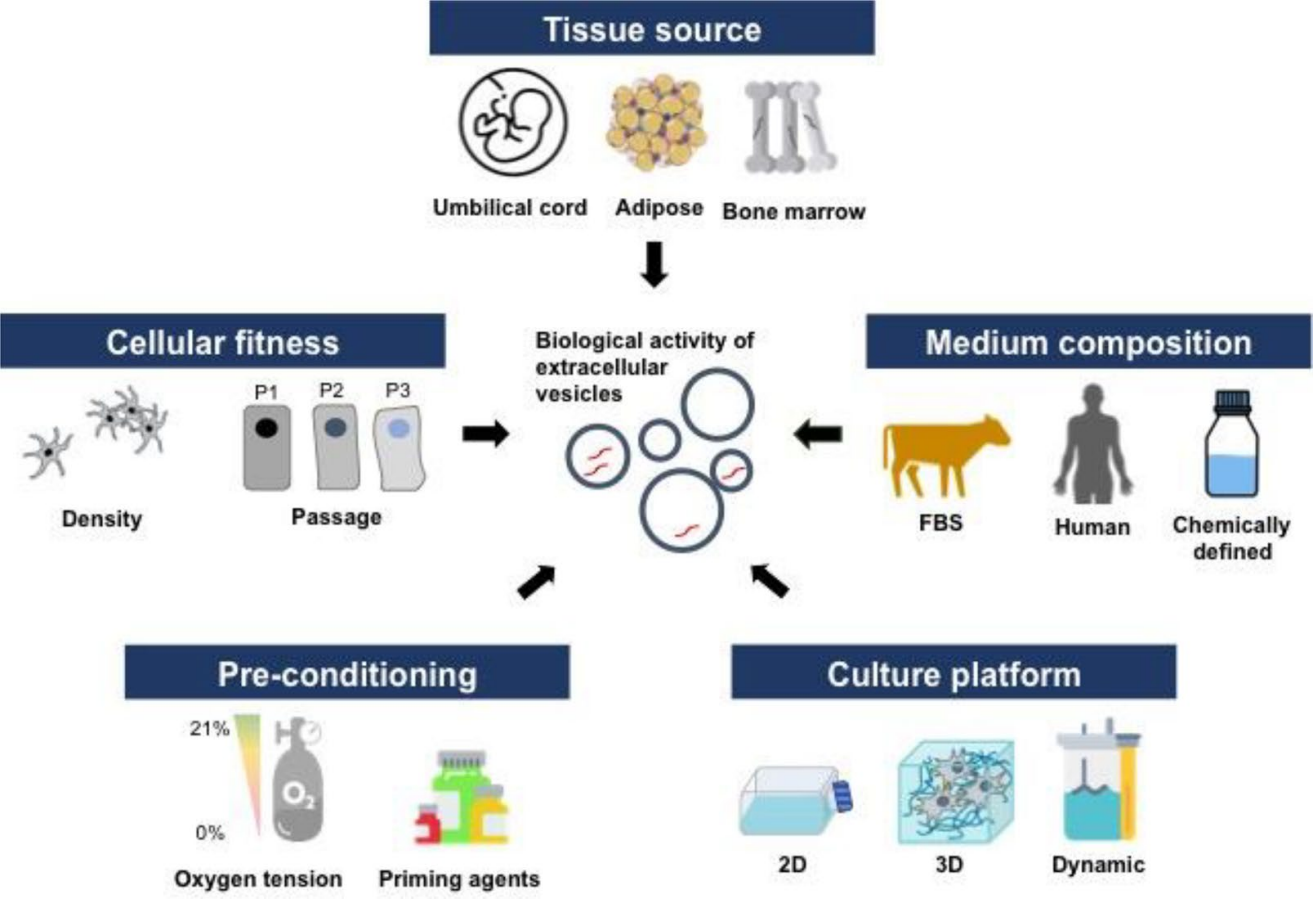
Key considerations for MSC-derived EV production. EVs are cellular products that are impacted by various culture conditions including tissue source, cell state (cellular passage, cell density during cultivation) as well as medium composition and culture platforms. Different culture conditions have been shown to influence the biological function of EVs. Therefore, careful considerations of these parameters are required upon manufacturing EVs for therapies

Mesenchymal stem cells (MSCs) are multipotent, nonhematopoietic adult stem cells that are characterized by their capability to differentiate into mesenchymal lineages such as chondrocytes, osteoblasts and adipocytes as well as non-mesenchymal lineages including hepatocytes and neuronal cell types. MSCs have the ability of colony formation, self-renewal, and secretion of trophic factors such as cytokines and growth factors, which play major roles in physiological and pathological processes. For these reasons, MSCs have been extensively used for wound healing and immunomodulation by administration and migration to the damaged site, engraftment and subsequent differentiation into the desired tissue [11]. Numerous clinical trials have been conducted using MSC as therapeutic agents to treat diseases, such as multiple sclerosis, osteoarthritis, cardiovascular disease (CVD), Alzheimer’s disease, kidney disease, diabetes mellitus, knee cartilage injuries, organ transplantation, and graft-versus-host disease (GvHD). By August 2021, the National Institutes of Health clinical trial database www.clinicaltrials.gov contained over 1,100 registered clinical trials in the category of stem cell therapies. There is solid evidence that MSCs exert their effects mainly through strong paracrine action on the neighboring cells via the secretion of trophic bioactive factors, such as growth factors, cytokines and chemokines [12]. In addition to these soluble factors, it has become evident that MSC-derived EVs are part of the stem cell secretome and play a major role in mediating the effects of stem cells [13]. Moreover, cell-free therapies using EVs could circumvent disadvantages associated with MSC therapies, namely low survival rate of cells upon adminstration, morphological changes during therapy, and the possibility of dedifferentiation into undesired tissue cell types [14–16]. A search on clinicaltrials.gov revealed that by September 2021 84 trials of EVs from different sources were registered worldwide (Fig. 3). However, only 4 were related to MSC-derived EVs (search term: mesenchymal stem cell-derived extracellular vesicles) and 16 to MSC-derived exosomes (search term: mesenchymal stem cell-derived exosomes), indicating the novelty and potential of this source (Fig. 4).

MSC-derived EVs have successfully been used to treat GvHD and are considered less immunogenic compared to their parent cells due to their lower content of major histocompatibility complex (MHC) molecules. These characteristics of MSC-derived EVs and their inability to form tumors make them strong candidates for cell-free therapy [17]. For example, MSC-derived EVs have been found to protect against myocardial ischemia (MI) [18], to reverse radiation toxicity [19], attenuate mitochondrial damage [20], and to enhance survival after acute kidney injury (AKI) through the transfer of MSC-EV specific miRNAs, such as hsa-let-7b and hsa-let-7 g miRNAs [21].

Furthermore, EVs could as well provide a natural alternative to standard drug delivery systems as they possess low immunogenicity and cytotoxicity. Nanoparticle-based drug delivery systems based on polymeric micelles, liposomes and nano-sized polymer-drug conjugates serve to improve the pharmacokinetics and biodistribution of chemical and biological therapeutic agents [22]. Their application, however, is associated with concerns regarding their potential immunogenicity and cytotoxicity and their rapid clearance upon clinical administration [23, 24]. The protein and RNA in EVs are encapsulated by a lipid layer, providing a protective barrier, which increases the success rate of delivery to the target cells [25, 26]. Indeed, numerous studies indicate the efficiency of MSC-derived EVs as carriers of chemotherapeutics [27], as well as RNA-based- [28] and anti-inflammatory drugs [29]. Different uptake mechanisms have been proposed in the literature, including phagocytosis or fusion of EVs with the plasma membrane of recipient cells. In addition, cells might permit the selective uptake of EVs depending on their surface receptor repertoire [18].

The undeniable potential of MSC-derived EVs in regenerative medicine leads to new possibilities and growing interest of the scientific community [13]. However, despite the therapeutic promise and success of MSC-derived EVs, the use of these EVs in clinical settings will require the resolution of several critical issues, such as (i) large-scale production and isolation methods, (ii) methods for rapid and accurate quantification and characterization of EVs, (iii) precise characterization of the cargo, (iv) pharmacokinetics, targeting and transfer mechanisms of EVs to the target sites, and (v) safety profiles to determine the optimal clinical dosage and possible toxicities upon repeated administration [30–32]. Furthermore, there is increasing evidence showing that the properties and biological functions of EVs are influenced by different manufacturing parameters such as cell source, culture conditions as well as enrichment protocols and characterization strategies [32].

Hence, this review provides a summary on the effects of various parameters, particularly upstream process parameters, on therapeutically relevant properties and biological functions of MSC-derived EVs. Additionally, several downstream process parameters, such as isolation methods and storage strategies, will be discussed as these methods are crucial for the improvement of the purity and yield of MSC-EVs.

## Influences of process parameters on the quality and heterogeneity of MSC-derived extracellular vesicles

### MSC sources for EV production

The composition of EVs is largely determined by the cell source and by the physiological state of their parent cells [30, 33]. Indeed, studies have shown that the secretome of BM-MSC-EVs highly inhibit the accumulation of inflammatory and apoptotic cell and mediates the maturation, proliferation and activation of B cells by exerting differential mRNA expression of relevant genes [34]. Whereas, umbilical cord-derived MSC-EVs (ucMSC-EVs) suppress oxidative stress in cisplatin-induced AKI by activating ERK1/2 pathway, promote angiogenesis for fracture healing and improve proliferation and migration of skin cells for wound healing [35]. Shekari et al*.* [36] summarized in a recent review article that bone marrow MSC (43% of all publications included in the systematic review, used as MSC source for EV derivation) were the preferred source of EVs in different disease categories, except for studies that involved the skin, liver and the vasculature as well as reproductive systems. Other MSC sources listed were placenta-derived EVs (Plac-MSC-EVs), adipose tissue MSC-EVs (AD-MSC-EV), pluripotent stem cell-derived MSC (Pluri-MSC-EVs), and derived from other tissue-derived EVs (TD-MSC-EV). Pluri-MSC-EVs were prevalently used for treatments of the liver, inflammation, transplantation and musculoskeletal diseases, which could be related to their low immunogenicity compared to MSCs from other sources. Contrarily, AD-MSC-EVs were not widely used in cancer or pancreatic diseases, but rather for treatment of skin and inflammation and transplantation diseases. Interestingly, Plac-MSC-EVs were used for a diversity of aforementioned disease categories except for autoimmune conditions [36]. La Greca et al. [37] reported differences in the proteome profile of iPSC-derived, iPSC-MSC-derived (PD-MSC) and MSC-derived EVs. Apparently, iPSC-derived EVs share a greater number of proteins with their respective cells, as compared to PD-MSC-derived EVs. This suggests that upon differentiation from iPSCs to PD-MSCs, a change of the EV protein composition is mediated and therefore EVs from PD-MSCs acquire a more specific protein footprint and functionality related to the stem cell niche [37]. This indicates that MSCs from different sources, even from the same donor, indeed vary in their molecular composition as presented in Table 1. Consequently, these variations could therefore have influenced the functional differences as reported in the aforementioned studies. However, the authors of the respective studies did not discuss their choice of EV source for a particular disease model. Hence, further investigation needs to be performed to determine which MSC source for EV production is most suited for a particular disease. Besides the cell source, other parameters such as culture conditions, harvesting period, as well as enrichment methods impact the structural and functional EV heterogeneity [38, 39], which will be addressed in the following sections.

**Table 1:** Specific surface markers identified in purified samples from different MSC sources

	Harvest [hours]	EV marker							
		CD9	CD63	CD81	CD59	Alix	TSG 101	Hsp 70/90	
iPSC-MSC	72	+	+	+	+	−	−	−	Lai [14]
	24	+	+	+					La Greca [37]
	24	+	+	+		+	+		Zhao [40]
Adipose tissue	24	−	−	+	−	+	−	−	Otero-Ortega [41]
	24	+	+			+	+		Conolly [42]
	48		+			+	+		Zhu [43]
	24	+	+	+		+	+	+	Durcin [44]
	48	+	+						Eirin [45]
Umbilical cord	36	+	−	+					Zhang [46]
	24–48	+	+	+	+	−	−	+	Kilpinen [47]
	48	+	+	+	−	−	−	−	Wang [48]
	24	+		+					Zou [49]
	48	+	+			+			Zhang [50]
Bone marrow	24	−	+	+	+	−	−	−	Kim [51]
	72	+	+	+	+	−	+	−	Haraszti [52]
	7 days	−	+	−	−	+	+	−	Barile [53]
	24		+						Angulski [54]
	48	+		+					Shi [55]

### Upstream process parameters

The possibility to influence the EV phenotype by using different cell culture techniques might present a novel strategy for the production of “customized EVs” for cell-free therapy. However, uncertainties regarding certain characteristics, including the risk of teratoma formation, rapid clearance from blood after administration as well as their potential for hypertrophy, raise safety concerns and represent challenges for their translation into clinical application. Culture parameters including cultivation time, shear stress, oxygen supply, medium composition, as well as cell-material interactions have been shown to impact MSC characteristics, which subsequently affect the properties of released EVs [56].

#### Effects of exogenous serum-derived EVs

The composition of the culture medium appears to have an impact on EV production. Fetal bovine serum (FBS), human serum, or human platelet lysate (HPL) are crucial media supplements, but also constitute a major source of EVs and EV-like particles. Especially the use of FBS raises concerns as it may contain contaminating particles such as viral proteins, toxins and mycoplasma due to inconsistent manufacturing processes [57]. This, in turn, issues another challenge for the isolation of EVs, which will be further addressed in Sect. 3.3, as these particles are co-enriched in EV samples upon exposure to the cell culture [58]. In this context, HPL represents a superior serum alternative since the manufacturing processes are more controllable and provide consistent quality compared to FBS. Furthermore, as HPL allows for xeno-free culture of MSCs it facilitates the translation into clinical trials [59]. However, HPL also contains similar quantities of exogenous serum-derived EVs and other nanoparticles such as growth factors and protein aggregates. As of today, it is not evident that HPL-derived vesicles negatively impact the therapeutic functionalities of MSC-EV preparations obtained from HPL-containing culture medium. Hence, understanding whether exogenous serum EVs or EV-like particles support or counteract specific therapeutic effects of MSC-EVs is necessary. Nevertheless, as the composition of MSC-EV preparations is heterogeneous, the characterization and depiction of the distinct effects that are exerted specifically by MSC-EVs remain challenging. Despite this, about 83% of all registered studies have implemented serum-containing media [60].

Various protocols for the depletion of serum EVs or serum-free conditions have been proposed over the past few years to avoid this contamination with serum-derived EVs (Table 2). The use of EV-depleted FBS or human serum reduced cell growth-promoting activity in most cell types but enhanced growth upon supplementation with isolated FBS-derived EVs. In conclusion, exogenous serum EVs substantially influence the behavior of cultured cells [61]. In another study, human AD-MSC were cultured in EV-depleted medium and demonstrated similar proliferation rates and no significant differences in cell and EV morphology compared to the non-depleted serum medium [62]. Haraszti et al*.* reported an increase in the EV activity, in regards to siRNA transfer, despite the decrease of yield after serum deprivation of MSCs, which indicates that their biogenesis is differentially regulated under stress [63]. Hence, further investigations are needed to validate the impact of serum-free or EV-depleted medium towards the biological function of secreted EVs. Other medium-related parameters such as the presence of liposomes, calcium, and increased pH as well as the induction of oxidative stress have been reported to increase the EV production in different cell lines [64].

**Table 2:** Methods for the depletion of EVs in serum additives for cell culture medium

Method		References
Ultracentrifugation	120,000 g, 18 h, 4 °C, SW32 Ti rotor (Beckman Coulter, Brea, CA, USA)	[66]
Ultrafiltration	Amicon ultra-15 centrifugal filters (UFC910024, 100 K filters and benchtop Merk Millipore Ltd., Tullagreen, Carrigtwohill, Co. Cork, Ireland), 3,000 g, 55 min, 4 °C	[62]
Tangential flow filtration (TFF)	hollow fiber-modified polyethersulfone (mPES) membrane filter column (area 1,600 cm^2^, 500 kDa molecular weight cut off) operated on a KR2i TFF System (Repligen, USA)	[67]
Commercially available exosome-depleted serum or medium	MesenCult™-ACF Plus (STEMCELL Technologies, China);	[68]
	Exo-FBS™ (System Biosciences, Mountain View, CA, USA);	[69]
	Oxium^TM^EXO (patent No. PCT/CL2019/100175);	[70]
	RoosterCollect EV Pro™ (RoosterBio Inc., Frederick, MD, USA)	[71]
Fibrinogen and fibrin depletion	Hydrogel formation was facilitated for 4 h at room temperature (RT) followed by overnight incubation at 4 °C. The resulting coagulated medium was heated to 37 °C for 1 h to enable a complete fibrin clotting. Afterward, a collapse was induced by vigorous shaking followed by centrifugation at 3000 g for 10 min at RT. Finally, the clear medium supernatant was filtered through a 0.22 µm filter (Merck Millipore, Billerica, MA, USA)	[72]

In conclusion, certain factors need to be considered upon using serum-free or serum-depleted culture medium: (i) a switch to these medium compositions could cause an alteration of extracellular RNAs, (ii) starvation leads to a stress response of the cells, which could change distinct cellular processes and increase/decrease of EV production, and (iii) some components of serum may persist in the culture after changing to serum-free conditions [65].

#### Effects of 3D and bioreactor culture

The production of EVs has most commonly been performed in 2D tissue culture polystyrene flasks. However, planar surfaces do not represent the native microenvironment of cells, which affects the cellular behavior and, consequently, the nature of the cellular secretome. Recent findings show the cultivation of MSCs in a three-dimensional (3D) microenvironment provides continuous production of MSC-derived EVs with similar properties to in vivo EVs and enhanced therapeutic potential for different disease models. Indeed, MSC-derived EVs from 3D hollow fiber bioreactor (HFB) cultivation were superior to 2D MSC-EVs as they significantly improved renal function, attenuated inflammatory factors, and suppressed T cell and macrophage infiltration in a murine model of cisplatin-induced acute kidney injury [73]. Another study reported an increase of immunomodulatory cytokines including TGF-b1 and TLR4/NF-kB negative regulator let-7b-5p in MSC-derived EVs from a microcarrier-based (2.5D) cultivation in a spinner flask [74]. These findings suggest that 3D culture systems could facilitate MSCs to release more potent EV populations, in terms of their functionality.

Furthermore, the limited surface area provided in 2D flasks generates over-confluent cell monolayers, if not properly controlled. Patel et al*.* [75] reported density- and passage-related differences in the bioactivity of MSC-derived EVs. In this study, MSCs of different passage numbers were cultured in cell culture-treated flasks at distinct seeding densities. Vesicle collection from conditioned medium was performed after 24 h. High cell seeding densities (10^4^ cells/cm^2^) and passage number (> 5) resulted in reduced production per cell and diminished angiogenic bioactivity, while no significant differences were observed in regards to size (30–200 nm) and surface marker profiles. Increased MSC passage number was associated with alterations in genes involving cell cycle, protein ubiquitination, and apoptosis, all of which may result in decreased cellular activity [76]. It is thus likely that this diminished activity also impacts function, indicating that it is essential to maintain MSCs in a non‐senescent state to retain the therapeutic potential of MSC-derived EVs. As to the influence of cell density on EV release, the reduced release at higher seeding density could be due to metabolic effects. Additionally, it has been proposed that reduced cell–cell contacts at low seeding densities may also play a role in the observed increase in production, since EV release may be a compensatory intercellular communication mechanism. This is supported by the finding that the depletion of EVs from the culture microenvironment results in increased EV release, suggesting that continuous perfusion culture systems could increase the yield.

Another important factor is the harvesting period of EVs, which defines the period in which the cell is allowed to produce and release EVs into the culture medium. Common harvesting periods chosen by different groups range between 24 h and 7 days [77]. Lee et al. [78] reported an optimal harvesting period of 48 h for adipose tissue-derived MSC-derived EVs (adMSC-EVs), whereas Almeria and Weiss et al. [79] obtained the highest vesicle concentration after six days of adMSC culture, which included medium changes every other day. Overall, these studies highlight the need for careful consideration of the parameters of cell passage number and cell seeding density in the production of therapeutic EVs at laboratory scale as well as for the design of large‐scale manufacturing protocols.

The demand for high yields as a prerequisite for potential clinical applications of EVs requires novel culture strategies to scale up production and enhance the bioactivity of EVs. The use of dynamic, scalable culture systems has been promoted to meet this demand (Table 3). Furthermore, bioreactors enable continuous culture and monitoring of critical process parameters including O_2_ concentration and pH [80]. Currently, three main bioreactors are prominently used to produce high yields of MSC: (1) multilayer-stacked Cell-factories, (2) hollow fiber-based bioreactors, and (3) stirred-tank bioreactors [81]. These systems have garnered attention for EV production due to their successful expansion rate at large scales. While these systems have already been tested for MSC expansion, very few studies (less than 50 publications in PubMed using the search string “3D mesenchymal stem cell derived extracellular vesicles”) have yet been published regarding 3D MSC-derived EV production, warranting further investigations [65]. Cao et al. [73] reported that 2D and HFB-MSC-derived EVs did not differ significantly regarding their surface marker profiles, size, or morphology, however, an up to 19.4-fold increased yield was observed for HFB-MSC-derived EVs. Similarly, Yun et al*.* reported a 7.5-fold higher EV yield as well as enhanced therapeutic efficacy for HFB-MSC-EV as compared to 2D MSC-EVs [82]. Whereas, the cultivation of hUC-MSCs in a microcarrier-based culture was demonstrated to increase the yield of EVs up to 20-fold compared to 2D cultures [52]. Additional studies describe similar findings with culture systems such as a 3D-printed scaffold-perfusion bioreactor [83, 84], spheroid/aggregate/organoid culture [85], and 2.5D surfaces (e.g. microcarriers).

**Table 3:** Bioreactor systems for MSC-EV production

In vitro system	Origin of EVs	Yield	Harvest time	Medium supplement	Study
10-layer Nunc™ EasyFill™ Cell Factory™ (2D) systems (Thermo Fisher Scientific, USA)	UC-MSC	1.36 × 109 ± 3.49 × 108 up to 5.96 × 109 ± 7.11 × 108 particles/mL	48 h over 6 days	OxiumTMEXO	[70]
Quantum (3D) bioreactor culture system (Terumo BCT, USA)	BM MSC-derived Evs	1.04 × 1010 particles/mL	48 h over 12 days	α MEM supplemented with 1% L-glutamine, 5% human platelet lysate, and 1% penicillin-strep- tomycin	[31]
Microcarrier-based (2.5D) culture in stirred tank bioreactor	UC-MSC	27-fold	48 h	serum-/xenofree StemPro medium (A1067501; Life Technologies, USA)	[52]
Microcarrier-based (2.5D) cultivation in spinner flask	hBM-MSC	1 × 1011 particles/mL	48 h over 7 days	5% fetal bovine serum (FBS)	[74]
Hollow fiber (3D) bioreactor (Fibercell Systems, USA)	hBM-MSCs	5.5 × 1010 particles/mL	24 h over 25 days	RoosterCollect-EV ser-/xeno-free medium (RoosterBio Inc., cat.#M2001)	[71]
Microcarrier-based (2.5D) cultivation in Vertical-Wheel™	AD-MSC	3.1 ± 1.3 × 1011	48 h	DMEM low glucose, 5% v/v UltraGRO™-PURE, Antibiotic–Antimycotic 1x	[87]
	BM-MSC	2.8 ± 0.1 × 1011			
	UC-MSC	4.1 ± 1.7 × 1011 EV particles			

Overall, the available publications demonstrate an increased potential of 3D and dynamic culture systems towards improved yield and bioactivity. Appropriate adjustments of related bioreactor parameters such as oxygen supply, hydrodynamic shear stress, metabolic byproducts and pH balance are required as they differ for each cell type. Furthermore, standardization of protocols is required to progress into translational studies [86].

#### Pre-conditioning with cytokines and hypoxia

MSC-derived EVs contain factors that promote tissue regeneration by immunomodulation [88] and enable targeted therapies via the introduction of genetic information, such as miRNAs [18]. MSCs have been investigated and applied in cell-based therapy for years due to their immunomodulatory, inflammatory, and regenerative capacity. To enhance their therapeutic efficacy, priming of MSCs with cytokines, pharmaceutical drugs, or further culture conditions was investigated [89]. The efficiency of MSCs in affecting immunomodulatory processes is known to be altered by their extracellular environment, which translates into the MSC secretome including EVs [89]. Similar to cellular priming, EVs can be pre-conditioned to exhibit increased efficacy upon certain biological functions. Interestingly, priming, by both cytokines and hypoxia, influenced on the yield, cargo, and surface markers of MSC-derived EVs, but did not significantly influence their size and morphology [90].

EV production seems to increase upon stimulation with different cytokine mixtures that drive a specific response or force the expression of certain genes by the producer cells [91]. Several studies investigated the effect of inflammatory stimulation, including pro-inflammatory treatment with IFN-γ, TNF- α, and IL-1 on the immunomodulatory efficacy and therapeutic applicability of primed MSC-derived EVs.

**Fig. 5:**
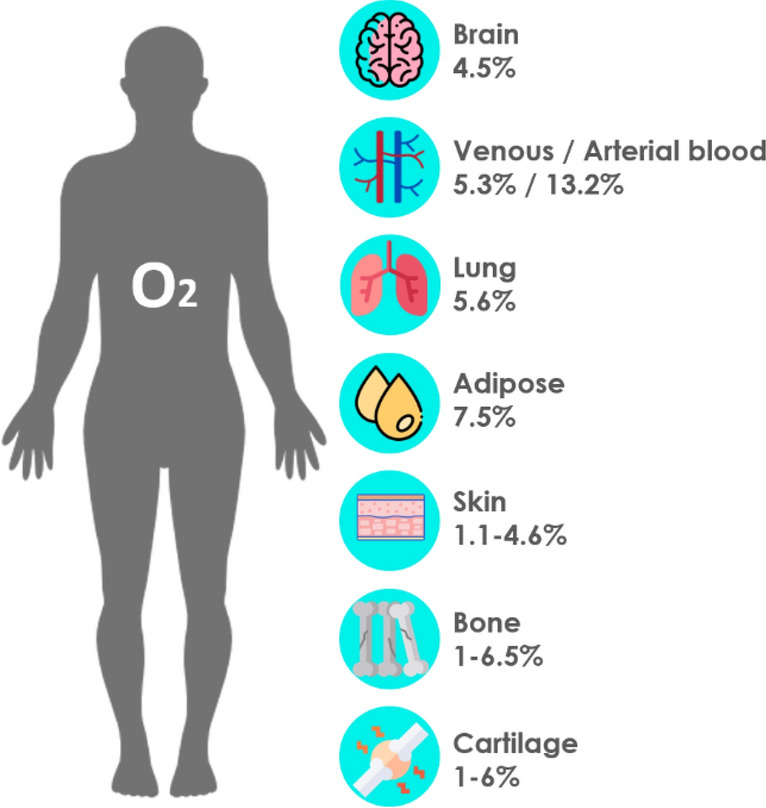
Physiological oxygen (O_2_) concentrations in different tissues. Illustration adapted [93]

Moreover, there is convincing evidence for the effectiveness of hypoxia-preconditioned MSC-derived EVs in immune modulation. Oxygen concentration regulates hypoxia-inducible factor-1 (HIF-1)-mediated transcription of various genes, such as VEGF, fibroblast growth factor 2 (FGF-2), hepatocyte growth factor (HGF), and insulin-like growth factor 1 (IGF-1), which maintain the stem cell fate in terms of proliferation and differentiation [92]. Hypoxia (1–10% O_2_) [93] is common in various adult human tissues as depicted in Fig. 5. Contrary to the MSC niche, which has been reported to reside at physiological O_2_ concentrations of 2–9%, standard laboratory conditions involve an ambient (normoxic) O_2_ level of 20% [94]. Therefore, comparative studies on the impact of normoxic and hypoxic conditions towards MSC functionality have emerged, and many of these studies have reported markedly different patterns of gene regulation under hypoxic cultivation of MSCs [95]. Indeed, hypoxia-preconditioning was observed to alter properties of MSC-derived EVs and to effect enhanced secretion, compositional changes of bioactive molecules [96], improved immunomodulation [97], angiogenic potential [79], reduction of reactive oxygen species (ROS), intracellular adenosine triphosphate (ATP) recovery, as well as inhibition of apoptosis [98]. MSC-derived EVs produced under hypoxic conditions showed an increase in proteins associated with chemotaxis (e.g. CCL3, MCP2, MCP4 and CSF-1) and angiogenesis, and the expression of CD9 and CD81 was statistically higher in hypoxic-conditioned EVs (*p* < 0.05) [99]. Similarly, those effects could be replicated by HIF-1 overexpression in normoxic cultured MSCs [100]. Bian et al. observed that the generation of human BM-MSC-EVs under hypoxia (1% O_2 _for 72 h) resulted in an improved cardiac regeneration in a rat myocardial infarction model by increasing angiogenesis at the infarcted area [101], supporting the potential of hypoxic preconditioning for regenerative applications [102].

#### Isolation and purification methods

EVs overlap in size and density with each other as well as with cellular components and organelles, including mitochondria [103]. On top of the diverse composition and function of EV subpopulations, such as exosomes or microvesicles, recent findings indicate that EV subpopulations released from different areas of the same cell (apical and basolateral EV) differ regarding their protein composition [56]. The distinction of populations and the designation of biological functions to individual populations—critical aspects for their potential therapeutic application—remains a challenge.

Protocols for sample preparation and MSC-EV enrichment influence not only the quantity but also the quality of EVs. Common isolation methods are based on physico-chemical properties of MSC-EVs, such as their density and size, or on the interaction with EV surface proteins (Table 4) [104, 105]. Ultracentrifugation at 100,000–200,000×*g*, has been used as the “golden standard” EV isolation method for many years, was reported to damage and disintegrate EVs due to the high *g* forces. Furthermore, sample viscosity, centrifugation time, as well as the rotor type (swing-out *vs.* fixed angle) affect EV isolation by centrifugation. Another disadvantage is that EV isolates could still be contaminated with proteins, which makes them unuseful for clinical application [104, 106]. Various studies have compared the different isolation techniques for MSC-EVs regarding criteria such as the resulting vesicle concentration and yield, size distribution, surface marker profiles, as well as the functional activity of the isolated MSC-EV populations [104, 107, 108]. Recent studies reported higher purity and functionality of MSC-EVs isolated by SEC rather than differential centrifugation (dUC). Nevertheless, the bottleneck includes high labor intensity and complete clearance of co-contamination with protein aggregates as well as lipoproteins is still not ensured [109, 110].

**Table 4:** Common isolation protocols used for MSC-derived EVs

Method		References
Differential centrifugation (dUC)	Prior to the ultracentrifugation (100,000–200,000 × g, 1-2 h, 4 °C) several low to intermediate-speed centrifugation steps are required to remove cells, cell debris, apoptotic bodies, and aggregates: 300–400 × g for 10 min sediment cells 1500–2000 × g for 15–20 min. at 4 °C remove cell debris 10,000 × g 15–30 min at 4 °C removal of other structures with a higher buoyant density that MSC-EVs	[79]
Density gradient isolation	Hereby, a continuous density gradient is formed by layering different concentrations of iodixanol. The MSC-EV-rich conditioned medium (CM) is overlaid on top and subjected to high-speed centrifugation (100,000 × g, 18 h, 4 °C), resulting in gradient fractions containing EV-like vesicles of different concentrations. Subsequently, these fractions are further processed in another high-speed centrifugation step (100,000 × g, 1-2 h, 4 °C) to separate MSC-EVs from other proteins and nucleoproteins	[111]
Size-exclusion chromatography (SEC)	CM is concentrated using a 100 kDa molecular weight cut-off filter to reduce total volume prior to the loading onto the column. The most common stationary phase used for EV isolation using SEC is Sepharose CL-2B, which is extensively washed and then packed into a column or syringe. The CM is loaded on top and EV-rich fractions are collected immediately and pooled after elution and again concentrated for further analytical procedures	[109]
Precipitation/Phase separation	The majority of protocols use polyethylene glycol (PEG)-based volume exclusion which precipitates EVs to a pellet. Hereby, CM is centrifuged at intermediate speed (6,000–10,000 × g, 45 min, 4 °C), filtered (0.22 µm), added to PEG solution to a final concentration of 10% (or 75 mM), and incubated for 8–16 h at 4 °C. Subsequently, the suspension is centrifuged and the EV-rich pellet is washed a few times with 0.9% NaCl. Lastly, the suspension is ultracentrifuged (100,000 × g, 130 min, 4 °C) and the resulting pellet is dissolved in buffer	[112]

Kamei et al. recently compared phosphatidyl serine (PS) affinity-based method (MagCapture Exosome, isolation Kit PS), polymer precipitation (ExoQuick, Total Exosome Isolation Reagent, and Exo-PREP), and size-exclusion chromatography (SEC) (qEV column) for the isolation of MSC-derived EVs and found that size, protein content, and yield varied depending on the method of isolation. In summary, results from that study show the highest purity obtained from PS affinity method compared to the other methods described. However, the outcome was connected with high EV loss and saturation of EV binding to the MagCapture beads. These observations demonstrate a disadvantage for clinical translation using the PS affinity method. On the other hand, SEC resulted in high protein concentration in fractions 7–9, which indicates a more effective collection of MSC-derived EVs [113]. Overall, the difficulty in isolating MSC-EVs in high yield or purity remains due to their small size and physicochemical heterogeneity. Hence, there is an urgent need to advance the technology to address this problem. Liangsupree et al*.* have recently summarized current and novel isolation techniques for EVs beyond ultracentrifugation and precipitation-based techniques [114]. The methods are categorized into (a) size-, (b) charge-, and (c) affinity-based techniques, which are listed in Table 5. Although most of these novel techniques have not been studied for MSC-EVs yet they represent promising approaches for the generation of highly purified MSC-EV isolates in the future.

**Table 5:** List of promising modern isolation and separation techniques for MSC-EVs [114]

Technique	Separation system	Advantages	Purity	Sample volume
Size-exclusion chromatography (SEC)	IZON® qEV column	Removal of co-contaminants including HDLs, albumin Yield better functionality of EVs compared to UC Less compositional and structural alterations comparted to precipitation techniques	+ + +	100 µl—10 ml
	Sepharose® CL-4B		+ + +	1 – 10 ml
Filtration-based	Centrifugal filter unit	Defined MWCO ranging from 10 – 100 kDa Simple and easy handling Cost- and time-effective	+	Up to 10 ml
	Tangential Flow Filtration (TFF)	Higher concentration of EVs	+	> 10 ml
	Hydrostatic filtration dialysis (HFD)	No centrifugation step Low EV loss	+	> 10 ml
Flow field-flow fractionation	asymmetrical flow field-flow fractionation (AsFlFFF or AF4)	Cross-flow can be modified Optimization between runs possible to enhance separation efficiency More flexible compared to sec Gentle fractionation	+ +	> 10 ml
	Deterministic lateral displacement (DLD) pillar array	Enables separation of exosomes in the size range of 20 to 110 nm	+ +	> 10 ml
Charge-based	Anion-exchange chromatography (AIEC)	Shorter isolation time (< 3 h for 1 L of cell culture supernatant) Yield intact evs	+ +	Up to 1L
	Electrophoresis and dielectrophoresis (DEP)	Subpopulations separated based on electrophoretic mobilities acquire information on properties of charged and non-charged EVs	+ +	> 10 ml
Affinity-based	Magnetic beads	Highly selective and specific Isolate evs originating from different cell types	+ + +	100 µl–1 ml

#### Storage and logistics

Next to isolation, storage can cause alterations in functionality. Generally, samples should be processed immediately after collection to preserve the stability and integrity of the membrane vesicles and to avoid aggregation of the EV preparations [115]. Approaches for EV preservation include (i) cryopreservation [116], (ii) freeze-drying [117], and (iii) spray-drying [118]. Studies on long-term storage of EVs have reported temperatures of − 20 °C as the upper limit under which EV from human embryonic kidney (HEK) 293 T cells, endothelial colony-forming cells (ECFCs,) and MSCs remain stable [119], whereas the optimal mode of storage was in the range of − 80 to − 70 °C [120]. As of today, however, no general standards regarding sample storage and processing of preparations have been defined [121].

## Conclusion

The use of MSC-derived EVs instead of stem cells confers several advantages, such as an improved safety profile, lower immunogenicity, as well as the ability to cross biological barriers. Furthermore, potential complications, including stem-cell-induced tumor formation, entrapment in the lung microvasculature, or immune rejection may be avoided by using MSC-derived EVs [1]. Despite promising results in preclinical trials, the use of MSC-derived EVs in clinical settings requires the resolution of several critical issues, including large-scale production, standardized isolation, quantification, and characterization procedures for MSC-derived EVs. Furthermore, an enhanced understanding of their targeting mechanisms and pharmacokinetics, as well as the determination of the optimal clinical dosage is still ongoing [122]. These aspects represent key elements for a successful EV-based therapy preventing risks, such as potential side effects on healthy cells, uncontrolled biodistribution and targeting, limited loading capacity, and insufficient clinical-grade production [123]. In this review, we summarized upstream process parameters that crucially affect the therapeutic properties and biologic functions of MSC-derived EVs. Critical upstream process parameters are (i) cell source, passage number, seeding density and confluence, (ii) medium composition, (iii) choice of 3D culture method and bioreactor type, and (iv) pre-conditioning of cells with cytokines or hypoxia. Additionally, critical downstream process parameters including isolation, purification, storage strategy as well as the characterization of MSC-derived EVs need to be considered for the manufacturing of clinical-grade EVs. The use of three-dimensional microenvironments, including bioreactors, for large-scale MSC-derived EV production is increasing nowadays and indicates a more efficient approach compared to traditional two-dimensional cell culture [73, 87, 124]. Combinations of different isolation methods, such as SEC and ultrafiltration-based method, currently garner great attention as it demonstrates to provide both high yield and high purity of selective MSC-derived EVs for the desired application [110]. Moreover, all these process parameters have to be aligned and optimized towards each particular target treatment resulting in unique processes despite not a universally valid solution. Overall, MSC-derived EVs indicate great benefits for biomedical applications, however, still significant challenges remain. Hence, continuous development and optimization of technologies are required to achieve higher efficiency and/or purity for the production and isolation of clinical-grade MSC-derived EVs.

## Data Availability

No applicable.

## Abbreviations

EVs: Extracellular Vesicles
MSC: Mesenchymal stem cell
MVB: Multivesicular body
RNA: Ribonucleic acid
mRNA: Messenger ribonucleic acid
FasL: Fas ligand
CVD: Cardiovascular disease
GvHD: Graft-versus-host disease
MHC: Major histocompatibility complex
MI: Myocardial ischaemia
AKI: Acute kidney injury
iPSC: Induced pluripotent stem cell
PD-MSC: Induced pluripotent mesenchymal-derived stem cell
Plac-MSC-EVs: Placenta-derived EVs
AD-MSC-EVs: Adipose tissue-derived MSC-EVs
TD-MSC-EVs: Tissue-derived EVs
BM-MSC-EVs: Bone marrow-derived MSC-EVs
ucMSC-EVs: Umbilical cord-derived MSC-EVs
FBS: Fetal bovine serum
HPL: Human platelet lysate
siRNA: Small interfering RNA
RT: Room temperature
3D: Three-dimensional
HFB: Hollow fiber bioreactor
TGF-b1: Transforming growth factor beta 1
NF-κB: Nuclear factor kappa-light-chain-enhancer of activated B cells
miRNA: Micro RNA
IFN-γ: Interferon gamma
TNF-α: Tumor necrosis factor
IL1: Interleukin-1
HIF-1: Hypoxia-inducible factor-1
VEGF: Vascular endothelial growth factor
FGF-2: Fibroblast growth factor 2
HGF: Hepatocyte growth factor
IGF-1: Insulin-like growth factor 1
ROS: Reactive oxygen species
ATP: Adenosine triphosphate
CCL3: Chemokine ligand 3
MCP2: Monocyte chemoattractant protein 2
MCP4: Monocyte chemoattractant protein 4
CSF-1: Colony stimulating factor 1
PBF: Phosphate buffered saline
dUC: Differential ultracentrifugation
SEC: Size-exclusion chromatography
MWCO: Molecular weight cut-off
HEK 293T: Human embryonic kidney 293T cells
EFCFs: Endothelial colony forming cells
